# A new perspective on mesenchymal stem cell-based therapy for liver diseases: restoring mitochondrial function

**DOI:** 10.1186/s12964-023-01230-0

**Published:** 2023-08-18

**Authors:** Yelei Cen, Guohua Lou, Jinjin Qi, Min Zheng, Yanning Liu

**Affiliations:** The State Key Laboratory for Diagnosis and Treatment of Infectious Diseases, National Clinical Research Center for Infectious Diseases, Collaborative Innovation Center for Diagnosis and Treatment of Infectious Diseases, The First Affiliated Hospital, College of Medicine, Zhejiang University, 79# Qingchun Road, 6A-17, Hangzhou, 310003 China

**Keywords:** Mesenchymal stem cell, MSC therapy, Liver disease, Mitochondria, Mitochondrial dysfunction

## Abstract

Mesenchymal stem cells (MSCs) have emerged as a promising alternative treatment for liver disease due to their roles in regeneration, fibrosis inhibition, and immunoregulation. Mitochondria are crucial in maintaining hepatocyte integrity and function. Mitochondrial dysfunction, such as impaired synthesis of adenosine triphosphate (ATP), decreased activity of respiratory chain complexes, and altered mitochondrial dynamics, is observed in most liver diseases. Accumulating evidence has substantiated that the therapeutic potential of MSCs is mediated not only through their cell replacement and paracrine effects but also through their regulation of mitochondrial dysfunction in liver disease. Here, we comprehensively review the involvement of mitochondrial dysfunction in the development of liver disease and how MSCs can target mitochondrial dysfunction. We also discuss recent advances in a novel method that modifies MSCs to enhance their functions in liver disease. A full understanding of MSC restoration of mitochondrial function and the underlying mechanisms will provide innovative strategies for clinical applications.

Video Abstract

**Supplementary Information:**

The online version contains supplementary material available at 10.1186/s12964-023-01230-0.

## Background

Liver disease notably contributes to the global burden of disease and mortality. The past few years have witnessed an increase in liver-related mortality worldwide from 3% in 2000 to 3.5% in 2015 [1]. Liver transplantation is the only effective treatment available for end-stage liver disease. However, the use of liver transplantation is limited by high costs and a shortage of donors. MSCs are self-renewing cells that can be obtained from a variety of tissues, such as bone marrow, umbilical cord blood, peripheral blood, the synovial membrane, and adipose tissue [2]. In terms of clinical use, MSCs have several advantages over conventional therapeutic approaches, such as their ease of harvesting, multilineage differentiation potential, and powerful immunosuppressive effects [3–5]. Therefore, MSCs have emerged as a promising candidate for novel cell-based therapies for liver disease.

Significant curative effects of MSCs have been observed in animal models of various liver diseases, such as cirrhosis and liver failure, as well as in clinical studies [4]. To account for the potential mechanism by which MSC treatment affects liver disease, early studies have shown that MSCs can be manipulated for differentiation into hepatocyte-like cells in vitro and in vivo [6]. Then, the robust immunoregulatory effects of MSCs through direct interaction with various immune cells or the production of a series of growth factors, cytokines, and signaling molecules have been observed [4]. Recently, a growing number of studies have shown that MSCs can modify mitochondrial dysfunction in acute liver injury and chronic liver disease, resulting in significant therapeutic effects [7–10].

Mitochondria are regarded as the powerhouse of the cell and drive and maintain highly organized cellular activities [11]. Similarly, mitochondria are the main energy source of hepatocytes and play a major role in the extensive oxidative metabolism and normal functions of the liver. Mitochondria are also central to the survival signaling pathways involved in liver injury [12]. Through in-depth exploration of the mechanisms of liver disease, researchers have discovered that mitochondrial dysfunction plays a significant role in the development of diseases such as alcohol-associated liver disease (ALD) [13], nonalcoholic fatty liver disease (NAFLD) [14], drug-induced liver disease (DILD) [15], viral hepatitis [16], cirrhosis [17], hepatocellular carcinoma (HCC) [18], and liver ischemia/reperfusion (I/R) injury [19]. Increasing evidence suggests that MSCs play a role in restoration of mitochondrial function in liver disease [20–22]. For example, MSCs can ameliorate lipid load by transmitting mitochondria to hepatocytes [8].

In this review, we summarize the latest findings on MSC-mediated restoration of mitochondrial function in liver disease from multiple perspectives, including mitochondrial transfer, restoring oxidant/antioxidant balance, regulating the mitochondrial quality control system, and improving lipid metabolism and calcium homeostasis. We also propose novel strategies to improve the efficacy of MSC therapy by modifying MSCs to focus their regulatory abilities on improving mitochondrial function. Therefore, this review may help to refine the understanding of the mechanisms of MSC therapy and provide novel strategies for MSC-based treatment of liver diseases.

## Mitochondrial dysfunction and liver diseases

### Overview of mitochondria and their functions

Mitochondria are small organelles with phospholipid bilayer structures. Because of this double-membraned structure, mitochondria are divided into five different regions: the outer mitochondrial membrane (OMM), the intermembrane space, the inner mitochondrial membrane (IMM), the cristae space, and the matrix. Each of these regions has a highly distinct functional role. For example, the highly folded IMM contains protein complexes of the electron transport chain (ETC). The number of mitochondria in cells varies widely by cell type. A mature red blood cell has no mitochondria, whereas hepatocytes can contain more than 2000 mitochondria [11]. Mitochondria have their own set of DNA as well as transcription and translation mechanisms. In mammals, mitochondrial DNA (mtDNA) is a single circular chromosome containing 37 genes that encode a number of proteins. mtDNA is vulnerable to endogenous and environmental damage [23].

A dominant function of mitochondria is to produce ATP, the main energy-carrying molecule of living cells. This process takes place through the tricarboxylic acid (TCA) cycle and oxidative phosphorylation (OXPHOS). In brief, acetyl-CoA derived from carbohydrates, fats, and proteins is oxidized via the TCA cycle in the mitochondrial matrix to produce NADH, FADH_2_, and other high-energy molecules [24]. Then, electrons from NADH and FADH_2_ are transferred to oxygen via the ETC, and a number of protons are pumped out of the mitochondrial matrix [25]. Subsequently, chemiosmosis occurs when the movement of protons establishes a strong electrochemical gradient across the inner membrane. Ultimately, the protons return to the mitochondrial matrix through the ATP synthase complex, and their potential energy is used for ATP synthesis. Together, the electron transport chain and chemiosmosis make up OXPHOS [26].

Mitochondria are not only the sites of ATP synthesis but also a resource for biosynthesis and cell signaling. First, mitochondria are the primary producers of superoxide anion radical (O_2_• –), which is a form of reactive oxygen species (ROS). In normal cells, approximately 0.1–2% of electrons contribute to producing O_2_• –  via complex I and complex II of the ETC. In addition, ROS include hydrogen peroxide (H_2_O_2_) and other oxidants derived from molecular oxygen. ROS are generated by NAPDH oxidases, mitochondria, endoplasmic reticulum, peroxisomes, and external stimuli. Low levels of ROS are essential for the regulation of signaling pathways and redox homeostasis [27]. Second, mitochondria are highly dynamic organelles that remodel their shape, distribution, and size in response to different conditions. These changes are mediated by fission and fusion events, which are crucial for the cell cycle, apoptosis, and mitochondrial quality control [28]. Mitophagy is a type of autophagy that is conducted by selective elimination of damaged mitochondria and maintains mitochondrial quality control [29]. Third, mitochondria are structurally and functionally related to the endoplasmic reticulum (ER). Both mitochondria and the ER are important organelles for the storage and buffering of calcium. Calcium homeostasis is required for muscle contraction and the activation of a series of second messenger system proteins. However, an increase in mitochondrial Ca^2+^ promotes cell death by triggering necrosis, apoptosis, and autophagy [30]. Moreover, the ER and mitochondria regulate lipid homeostasis together. Some lipids in the ER, such as phosphatidylserine, need to be transferred to the mitochondrial inner membrane, where they are converted to phosphatidylethanolamine by mitochondrial lipid processing enzymes. Mitochondria also serve as the place where fatty acids are broken down to generate acetyl-CoA through β-oxidation [11]. Recently, it has been found that the interface between lipid droplets and mitochondria in hepatocytes allows free fatty acid (FFA) transfer from storage to utilization, which provides an efficient way to avoid lipotoxicity [8].

In conclusion, mitochondria are indispensable for energy production, signaling, programmed cell death, and many other metabolic tasks in all cell types. If any portion of this precise organelle is impaired or depleted, it will have an impact on physiological activity and expedite the progression of disease.

### Mitochondrial dysfunction also exists in many liver diseases

**Fig. 1:**
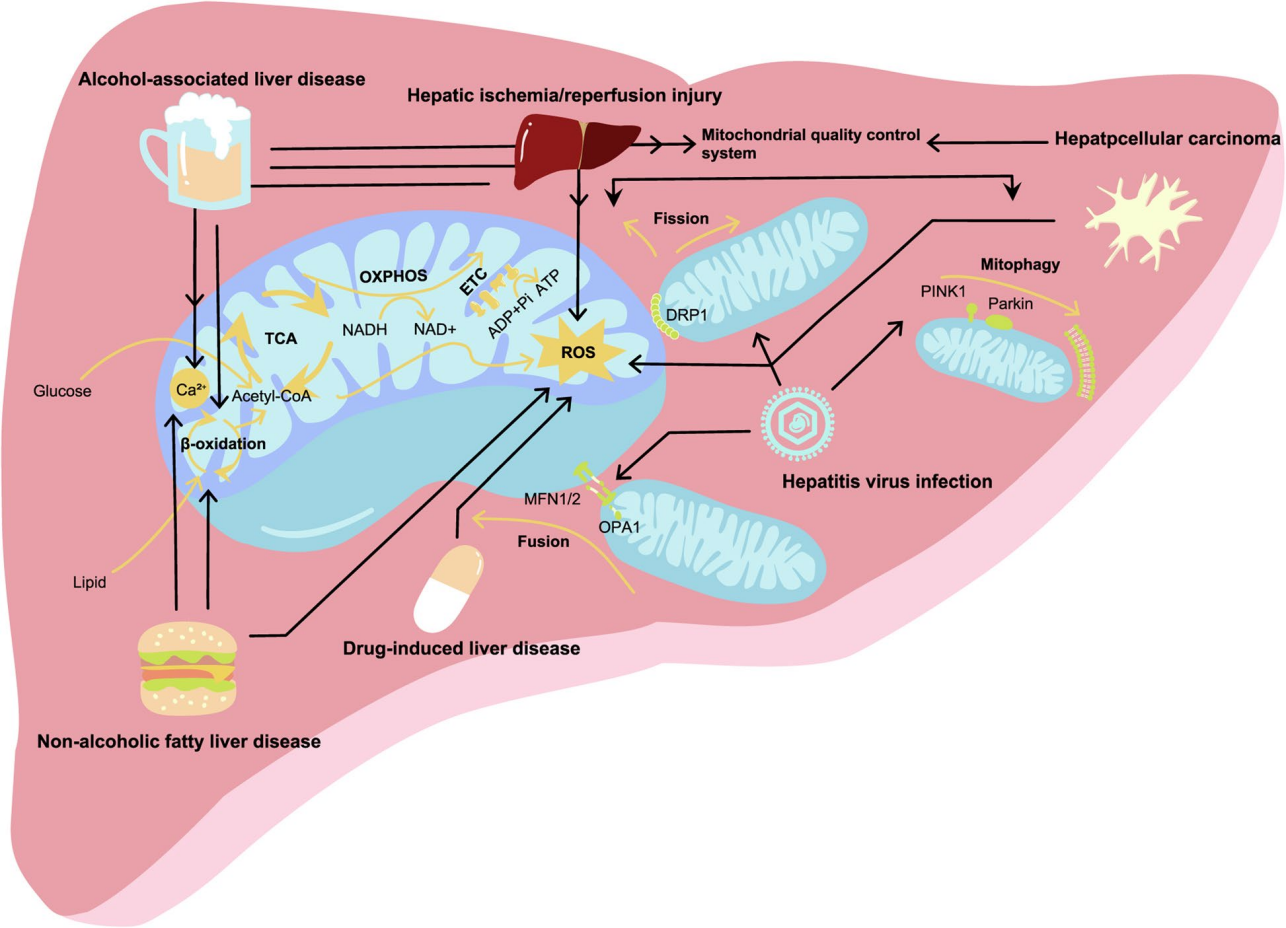
Common mitochondrial dysfunctions in liver disease. In the liver, mitochondria are responsible for energy production and biological metabolism. ROS is a product of OXPHOS and excessive ROS causes damage to the mitochondria, which is observed in almost all liver diseases. In ALD and NAFLD, disturbances in lipid metabolism and calcium homeostasis are common mitochondrial dysfunctions. The mitochondrial quality control system maintains healthy mitochondrial turnover, which is driven by complex pathways involving DRP1, MFN1/2, OPA1, PINK1, and Parkin. An impaired quality control system is observed in ALD, hepatic I/R injury, hepatitis virus infection, and HCC

The liver is the central organ for the homeostasis of carbohydrate, lipid, and protein metabolism. Mitochondria in hepatocytes perform unique functions, such as regulating the gluconeogenic process, ammonia detoxification, and anabolic pathways, all of which are necessary for metabolic regulation [31, 32]. Therefore, dysfunctional mitochondria in hepatocytes can disrupt overall body homeostasis. The most common types of mitochondria dysfunction in liver disease are increased oxidative capacity and diminished antioxidant defense [33]. The disruption of oxidant-antioxidant balance is caused by the enhanced production of ROS or the depletion of antioxidants. The accumulated ROS can directly cause mtDNA and mitochondrial membrane damage. These factors further promote ROS production and ultimately, a vicious cycle is formed. Most liver diseases are associated with more than one type of mitochondrial dysfunction (Fig. 1).

#### Alcohol-associated liver disease

In ALD, ethanol can cause mitochondrial dysfunction in many ways. First ethanol is converted to acetaldehyde in liver by enzymes called alcohol dehydrogenase (ADH). This reaction consumes NAD + and produce NADH, increasing the NADH/NAD + ratio [34]. NADH leads to ROS formation and inhibits complete fatty acid oxidation in the liver via competitive substrate oxidation of the mitochondrial ETC [35]. Second, ethanol induces cytochrome P-450 2E1 enzyme expression in mitochondria, which promotes ROS generation [36]. Third, in patients with alcoholism, mtDNA undergoes oxidation, depletion, and deletion, which will cause mitochondrial dysfunction once a threshold sufficient to impair mitochondrial respiration and ATP synthesis is reached [17]. Moreover, alcohol intake will cause cholesterol overload in the mitochondria. Overloaded cholesterol will decrease membrane fluidity and impair mitochondrial glutathione (GSH) transportation,an antioxidant that maintains an appropriate mitochondrial redox environment [37]. In addition, alcohol-induced ER stress can indirectly alter GSH levels by increasing cholesterol synthesis and altering its translocation to mitochondria [17]. A novel pathway involved in ALD has recently been illustrated. Ethanol induces an upregulation of the DNA-dependent protein kinase catalytic subunit, which activates the mitochondrial fission mediated by dynamin-related protein 1 (Drp1) and inhibits protective mitophagy through the upstream transcription activator P53 [13].

#### Nonalcoholic fatty liver disease

NAFLD is a generic term for disorders characterized by an accumulation of excess fat in hepatocytes that is not caused by alcohol. Impaired mitochondrial function and morphology have been found in NAFLD patients. Lipid overload may increase oxidative metabolism but result in poor ATP production due to mitochondrial uncoupling. The adaptation of oxidative metabolism in response to lipid overload also leads to an impaired ETC, which causes the liver to bear high amounts of oxidative stress. The increased cardiolipin in the mitochondrial membrane can be transferred to the OMM and form pores, which leads to the release of cytochrome c into the cytosol and activates the apoptosis pathway [14]. The lipotoxic environment also induces ER stress and disrupts the structural integrity between the ER and mitochondria, which will lead to increased Ca^2+^ influx from the ER to the mitochondria, thus promoting ROS production and inducing the opening of the mitochondrial permeability transition pore (MPTP) [38].

#### Drug-induced liver disease

Since many drugs are metabolized in the liver, they have the potential to disturb mitochondrial function by targeting a certain step or enzyme in metabolism or energy production. For example, acetaminophen (APAP), a drug used to relieve pain and reduce fever, is metabolized by the liver to N-acetyl-p-benzoquinone imine (NAPQI). The accumulation of NAPQI alters mitochondrial proteins, such as voltage-gated ion channels, forming NAPQI protein adducts that increase ROS generation and decrease ATP production [15]. Moreover, APAP-induced excessive mitochondrial oxidative stress activates c-jun N-terminal kinase (JNK) in the cytosol and its translocation to the OMM, which leads to the inactivation of Src kinase, which maintains electron transport. Finally, this process reduces mitochondrial respiration and amplifies the formation of oxygen free radicals and peroxynitrite [39].

#### Viral hepatitis

Hepatitis B virus (HBV) and hepatitis C virus (HCV) infections are frequently accompanied by impaired ETC function and mitochondrial compartment rearrangement. Hepatitis viruses actively interact with ETC proteins. For example, the HBx protein and HCV replication can inhibit ETC activity and impair ATP production. Hepatitis viruses also have complex interactions with MPTP, a conductance channel that forms in the IMM in response to excessive cytosolic Ca^2+^ or ROS. The opening of mPTP leads to mitochondrial swelling and necroptosis [40]. HBV and HCV core proteins, for example, induce MPTP opening or blocking to regulate the release of mitochondrial contents, such as mtDNA fragments or ATP [16]. Both HBV and HCV infections increase the phosphorylation and translocation of Drp1, a self-assembly oligomer that mediates mitochondrial fission, resulting in the promotion of mitochondrial fission and mitophagy. Infection also provokes mitophagy by increasing the expression of PTEN-induced putative kinase protein 1 (PINK1) and Parkin, as well as Parkin mitochondrial translocation. These processes may inhibit the death of infected cells and act as a viral survival mechanism [12].

#### End-stage liver disease and liver ischemia/reperfusion injury

ALD, NAFLD, or viral infection can all develop into cirrhosis, which is caused by scar tissue replacing healthy liver tissue. In patients with cirrhosis, lipid and amino acid metabolism as well as mitochondrial dynamic function are impaired. Mitochondrial dysfunction is also actively involved in fibrogenesis and inflammation through the response to TGFβ, which is secreted by activated hepatic stellate cells and macrophages and is the major effector leading to hepatic fibrosis [17]. Cirrhosis is the most potent risk factor for the development of hepatocellular carcinoma. Excessive oxidative stress is caused by accumulated lipid droplets, which potentiate mtDNA mutations and reduce mtDNA copy number, both of which are common events that contribute to metabolic reprogramming and hepatocellular carcinoma progression [18]. Liver transplantation and resection surgery are effective treatments for end-stage liver disease. However, a common complication after surgery is hepatic I/R injury, which is initiated during hypoxia or anoxia and becomes more severe when oxygen delivery and tissue pH are restored. Hepatic I/R injury is closely associated with mitochondrial dysfunction, such as calcium overload, excessive oxidative stress, and defective mitophagy [19].

Multiple factors lead to mitochondrial dysfunction in different liver diseases. These factors will undoubtedly affect liver metabolism and detoxification, hastening the progression to end-stage liver diseases. It seems that increased ROS due to mitochondrial dysfunction is prevalent in liver diseases, which further causes severe dysfunction, such as mtDNA mutations. As a result, restoring mitochondrial function, such as delivering healthy mitochondria or repairing defective mitochondria, might be an innovative strategy for treating liver disease [41–43].

## MSCs exert curative effects in liver diseases by regulating mitochondrial function

MSCs have attracted great attention because of their outstanding performance in the treatment of liver diseases. During the last decade, it has been demonstrated that MSCs can treat liver disease by developing into hepatocyte-like cells and through their immunomodulatory capacity. Recently, the exciting outcome of transferring functional mitochondria from MSCs into mature cardiomyocytes provided a new idea for determining their therapeutic effects [44, 45]. Mitochondrial regulation is becoming one of the hottest topics in the investigation of MSC therapy mechanisms. A growing body of evidence from liver disease research suggests that MSCs can restore mitochondrial function. In this context, we will reveal potential mechanisms of MSC-mediated mitochondrial restoration in treating liver disease from the aspects of mitochondrial transfer, oxidation/antioxidant imbalance rectification, mitophagy and dynamic regulation, and lipid metabolism and calcium homeostasis modification (Table 1).

**Table 1:** Summary of the mitochondrial dysfunction modulation of MSCs in liver disease

**Liver disease**	**Mitochondrial dysfunction**	**Source**	**Delivery route**	**Potential mechanism**	**Ref**
NAFLD	Abnormal lipid metabolism and calcium homeostasis	Human bone marrow	Intrasplenic injection	Mitochondrial transfer via TNT	[8]
		Rat bone marrow	Tail vein	Restoring sarcoplasmic/ER Ca^2+^ ATPase activity to alleviate of ER stress	[46]
CCl_4_-induced liver injury	Oxidant/antioxidant imbalance	Human bone marrow	Tail vein	Increasing SOD activity and inhibiting ROS production	[47]
		Human umbilical cord	Tail vein	Presented more distinct antioxidant by EVs	[48]
APAP and H_2_O_2_-induced liver injury	Oxidant/antioxidant imbalance	Rat bone marrow	Intrahepatic injection	Fractionated MSC exosomes reduce ROS activity more efficiently	[49]
	Oxidant/antioxidant imbalance	Mouse adipose tissue	Tail vein	Increasing hepatic GSH level and alleviate ROS accumulation	[10]
D-galactose induced liver injury	Oxidant/antioxidant imbalance	Human umbilical cord	Tail vein	Reducing oxidative stress via activation of Nrf2/HO-1 pathway	[50]
Hepatic I/R injury	Oxidant/antioxidant imbalance	Human umbilical cord	Tail vein	Suppressing oxidative stress by MnSOD encapsulated in EVs	[21]
	Impaired mitophagy	Human umbilical cord	Peripheral vein	Reducing Parkin and PINK1 expression and inactivating AMPKα pathway	[22]
Post-hepatectomy liver failure	Impaired mitochondrial dynamics	Mouse bone marrow	Portal vein	Downregulating p-Drp1 and FIS1 expression and upregulating MFN2	[7]
	Abnormal lipid metabolism			Reducing mitochondrial damage and secreting IL-10	
Liver cirrhosis	Abnormal lipid metabolism	Human placenta	Tail vein	Attenuating ER stress via activating the EGFR-PI3K-CaM Pathway by PRL-1	[51]

### Mitochondrial transfer as a bridge in MSC therapy

To further investigate the effects of crosstalk between MSCs and other cells, fully differentiated mouse cardiomyocytes were cocultured with adipose-derived MSCs. For the first time, this study highlighted the critical function of mitochondrial transfer from stem cells to cardiomyocytes in somatic reprogramming [44]. Tunneling nanotubes (TNTs) and extracellular vesicles (EVs) are the most common methods used by MSCs to deliver mitochondrial cargo to other cells or injured tissue.

#### Tunneling nanotubes

TNTs extend from the plasma membrane and enable different mammalian cells to touch over long distances. TNTs were originally described as conduits through which mammalian cells could arrange the selective transfer of membrane vesicles and organelles between cells [52]. Currently, TNT-mediated transfer of mitochondria or mtDNA from MSCs to cells in other organs has been reported in respiratory, cardiovascular, neuronal, and immune system diseases and disorders [53].

When MSCs home to the liver, they can donate mitochondria to surrounding damaged hepatocytes through TNTs [54]. Recently, in a mouse model of NAFLD, MSCs reversed mitochondrial dysfunction by altering hepatic lipid metabolism from storage to utilization and ameliorated excessive oxidative stress associated with enhanced lipid oxidation. These effects were related to the delivery of mitochondria to mouse hepatocytes by TNTs [8]. Additional studies are needed to elucidate the mechanisms by which TNTs transfer functional mitochondria to injured cells.

#### Extracellular vesicles

EVs are lipid-bound vesicles produced in the endosomal compartment of most eukaryotic cells. The three main subtypes of EVs are microvesicles (MVs), exosomes, and apoptotic bodies, which are distinguished by their biogenesis, release pathways, size, content, and function [55]. MSCs have been shown to secrete an abundance of different types of EVs with proregenerative and anti-inflammatory effects in animal models of stroke, traumatic brain injury, and liver injury [56]. Therefore, MSC-EVs now have the therapeutic potential to go from laboratory to clinical trials for disease treatment.

Researchers have shown that MSCs can transfer mitochondria to impaired pulmonary alveoli in acute lung injury by releasing MVs [57]. Recently, an experiment showed that intramyocardial injection of MSC-EVs containing mitochondria could enhance cardiac function after myocardial infarction in vivo [58]. More study is needed to evaluate how mitochondrial donation of MSC-EVs occurs in the treatment of liver disease in comparison to what is known about TNTs. It is worth noting that MSC-EV transport of other mitochondrial cargoes has a robust effect on rescuing mitochondrial function, as explained in the following section.

### MSCs restore oxidant/antioxidant balance

The progression of energy production in mitochondria, such as the TCA cycle and fat metabolism, cannot occur without an oxidation reaction. As we described above, this reaction involves mitochondria and produces ROS such as O_2_• – and H_2_O_2_. Excessive O_2_• – and H_2_O_2_ can induce supraphysiological levels of oxidative stress [27]. Mitochondria are also equipped with antioxidants to quench oxidants. Manganese superoxide dismutase (MnSOD), for example, is an enzyme that converts excess O_2_• – to H_2_O_2_ and molecular oxygen. Mitochondrial antioxidants such as GSH and thioredoxin can buffer cellular H_2_O_2_.

MSC therapy has been observed to restore the oxidant/antioxidant balance. In a D-galactose-induced hepatitis rat model, MSC treatment could evoke the activation of the Nrf2 pathway and increase the downstream expression of heme oxygenase-1, an antioxidant that can translocate to mitochondria to reverse mitochondrial dysfunction [50]. MSCs transplantation significantly increased SOD activity and decreased ROS production in CCl_4_-induced liver injury [47]. Some researchers have shown that this effect might be achieved by MSC-EVs. Recently, a study showed that EVs from human umbilical cord MSCs (hUC-MSC-EVs) shuttled MnSOD to hepatic tissue after I/R injury. In this model, the knockdown of MnSOD in hUC-MSCs reduced their antiapoptotic and antioxidant effects [21]. The findings from another experiment in CCl_4_-induced liver injury indicated that hUC-MSC-EVs could suppress injury development via antioxidant effects more effectively than bifendate, a commonly utilized hepatic protectant [48]. These findings suggest that some components in MSC-EVs account for the mitochondria-mediated antioxidant and protective effects in liver disease. MSC-EVs may be more effective than antioxidant drugs.

### MSCs regulate the mitochondrial quality control system

In response to the stress and damage caused by disease, the mitochondrial quality control system is designed to identify and eliminate defective mitochondrial proteins, mitochondrial components, or entire mitochondria [59]. The quality control of mitochondria is achieved by mitophagy and mitochondrial dynamics, including fission and fusion. In many cells, mitophagy is regulated by parkin and PINK1. Mitophagy plays a key role in promoting mitochondrial turnover and preventing the accumulation of dysfunctional mitochondria [29]. Fission and fusion are closely related to the onset of mitophagy, and they help mitochondria separate from the mitochondrial network [60]. Another critical function of fission and fusion is to distribute mtDNA and proteins throughout the mitochondrial network, which optimizes mitochondrial function and prevents the accumulation of mitochondrial mutations during aging [61]. Fission in mammals is mediated by Drp1 which are recruited to the OMM from the cytosol through the outer membrane proteins such as mitochondrial fission factor, fission 1 (FIS1) and mitochondrial division factors 49 and 51 kDa. Mitochondrial fusion is mediated by several fusion proteins, including mitochondrion fusion-related protein 1 (MFN1), mitochondrion fusion-related protein 2 (MFN2), and optic atrophy 1 (OPA1). Some mitophagy receptor proteins interact with Drp1, MFN1/2, or OPA1 to facilitate mitochondrial dynamics [12].

Recently, UC-MSC treatment has shown a potential hepatoprotective effect in a liver I/R model by controlling mitochondrial quality. The underlying mechanism was that MSCs increased Parkin and PINK1 expression and mitophagy in injured tissue by activating the AMPK pathway [22]. The results from a model of posthepatectomy liver failure showed that MSC transplantation ameliorated the disruption of fission and fusion balance and protected the remaining liver from excessive lipid accumulation. This protective effect may be achieved through MSC secretion of interleukin 10 mediating the downregulation of increased phospho-DRP1 and FIS1 and the upregulation of MFN2 to preserve mitochondrial activity [7].

To date, little evidence on how MSCs regulate dynamics and mitophagy in hepatocytes has been found. In a recent experiment performed in a clinically related ARDS model, MSC-EVs containing mitochondria restored LPS-induced mitophagy to a normal level [62]. This finding shows that the regulation of the mitochondrial quality control system and mitochondrial transfer could serve as multifunctional effects of MSCs. Thus, we may reveal the complex mechanisms of MSC-based treatment in liver disease by learning from research in other diseases.

### Amelioration of lipid metabolism and calcium homeostasis in MSC treatment

As we discussed in earlier sections, mitochondria are where lipids are metabolized and cellular calcium is buffered, whereas the ER is the primary location for protein and lipid synthesis and cellular calcium storage. Mitochondrial calcium is mostly derived from the ER through the inositol 1,4,5-trisphosphate receptors (IP_3_R) channel in hepatocytes. Maintaining proper mitochondrial Ca^2+^ levels is needed to sustain liver metabolism by activating enzymes in the TCA cycle as well as lipid metabolism. Ca^2+^ homeostasis dysregulation can cause excessive ER stress and ROS production, and it can aid mitochondrial lipid uptake, which leads to altered lipid metabolism. This provides a unique viewpoint on the disease progression from NAFLD to HCC [63]. A recent study showed that a loss of the mitochondrial Ca^2+^ uniporter led to a restriction of Ca^2+^ buffering function in the mitochondria, which impaired OXPHOS activity and promoted hepatic lipid accumulation [64]. Thus, disturbances in lipid metabolism and calcium homeostasis are closely related to the dysfunction of mitochondria or the ER.

Several recent studies have shown that MSC treatment can be targeted to improve lipid metabolism and calcium homeostasis. In NAFLD rats, MSC treatment led to an improvement in lipid metabolism and an alleviation of ER stress. The molecular mechanism for this protective effect may be the minimization of calcium overload in the cytosol and ER by restoring the activity of a Ca^2+^ ion transporter called sarcoplasmic/ER Ca^2+^ ATPase [46]. Another study showed that MSCs could ameliorate ER stress-induced calcium imbalance and lipid accumulation in cirrhosis through another molecular mechanism, that is, by regulating the calcium channels between the ER and mitochondria and calmodulin-mediated calcium signaling [51]. Liver failure induced by hepatectomy is also related to abnormal lipid accumulation and mitochondrial damage. MSCs were found to elicit therapeutic benefits by secreting paracrine cytokines such as IL-10, which activated the mTOR-mediated cell metabolism regulatory mechanism, promoting fatty acid oxidation in mitochondria [7].

As various liver diseases are related to more than one kind of mitochondrial dysfunction, the therapeutic effect of MSCs on liver disease may occur through multifaceted modulation of mitochondrial function. Identifying the kind of mitochondrial dysfunction and describing the precise mechanisms of liver disease could provide further insight into how to improve the efficiency of mesenchymal stem cell therapy.

## MSC modification strategy based on the regulation of mitochondrial function

**Fig. 2:**
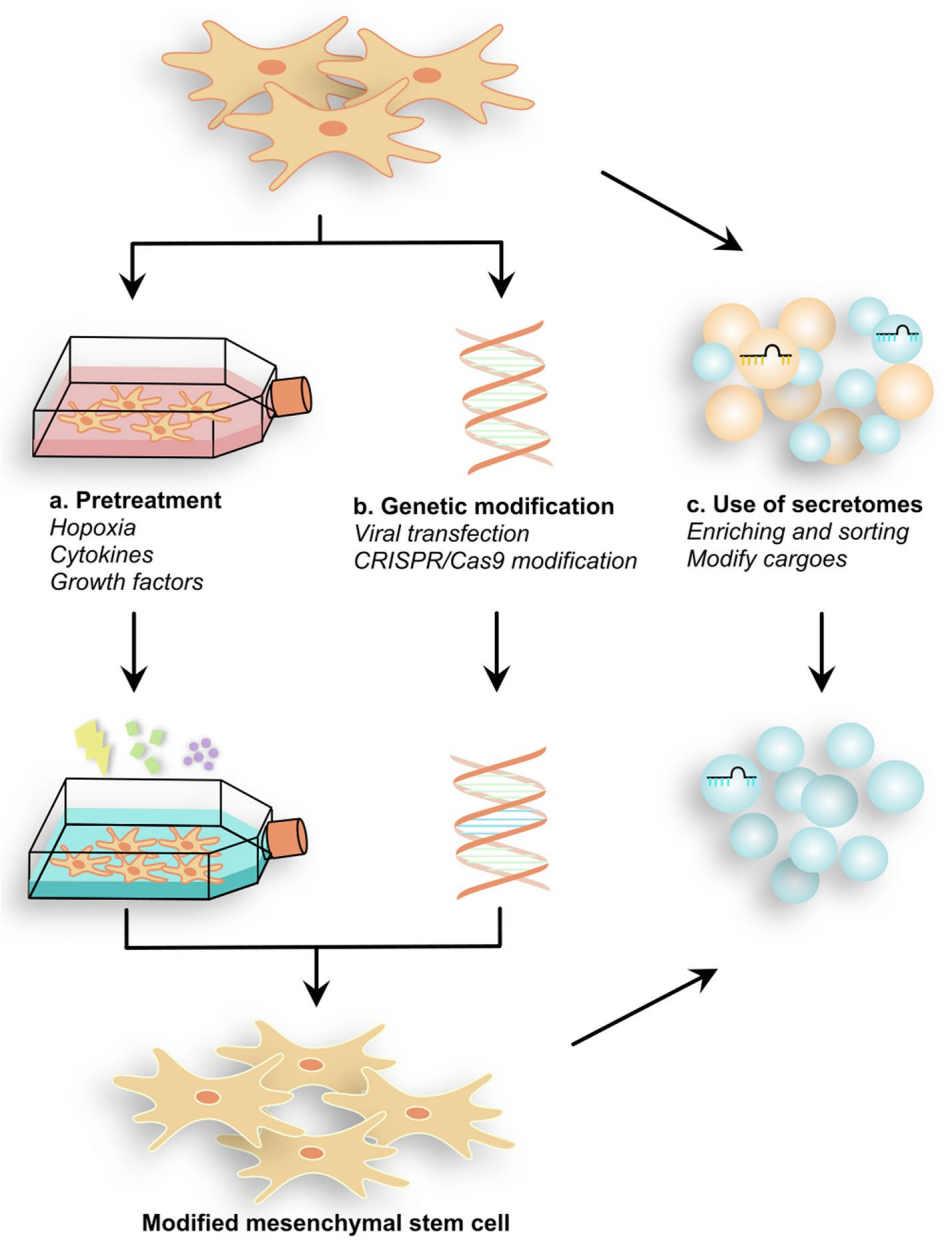
The modification method applied to MSCs. Three modification techniques to improve the inherent therapeutic properties of MSCs in remodeling mitochondrial function were developed. a. Pretreating MSCs with a specifically designed cultural environment will increase the rate of survival and enhance immunoregulation. b. Genetic modifications such as overexpressing antioxidant genes have reliable therapeutic effects against mitochondrial dysfunction. c. Using the secretomes derived from MSCs carrying functional carriers such as miRNAs that can specifically restore mitochondrial function. Pretreated and gene-modified MSCs could also exert their effects through secretomes

In addition to our previous studies on modified MSCs benefiting liver fibrosis [65] and HCC [66], modified MSC therapies have been noted to be effective in the treatment of other liver diseases as well. Currently, more precise modification methods for enhancing the effects of MSCs have emerged. These strategies may involve the priming of MSCs, the genetic modification of MSCs, or the use of secretomes from MSCs (Fig. 2).

First, pretreatment of MSCs with hypoxia, cytokines, chemical agents or different culture conditions has been shown to boost MSC survival and therapeutic effectiveness [67]. Hypoxia-pretreated MSCs were able to reduce supraphysiological oxidative stress levels and hepatocyte apoptosis in a CCl_4_-induced mouse cirrhosis model by increasing their own miR210 levels. This finding may imply that primed MSCs restore mitochondria better than unprocessed MSCs [68]. Second, genetic modification of MSCs, such as through the overexpression of antioxidant genes, might be another potential way to enhance the effectiveness of MSC therapies. MnSOD is a mitochondrial antioxidant enzyme that is regulated by the cell oxygen concentration. Once hepatic hypoxic-ischemic injury occurs, MnSOD is activated. The upregulation of the gene encoding MnSOD in MSCs by adenoviral constructs led to the amelioration of oxidative stress and apoptosis inhibition in liver I/R injury rats, demonstrating the hepatoprotective properties of the modified MSCs [69]. Finally, increasing evidence has confirmed that MSC-EVs are efficient cellular therapeutic agents owing to their advantages such as their ease of manufacture and administration, lack of tumorigenic side effects, and simple availability of high doses [56]. One strategy is enriching and sorting functional EVs to regulate mitochondrial function. Experiments conducted in an in vitro model of APAP-induced liver injury showed that the exosome-rich fractionated secretome of MSCs obtained by filter, concentration and ultracentrifugation is more effective in reducing excessive ROS activity than the unfractionated secretome. The findings from this study supported the hypothesis that soluble factors enriched in the fractions of exosomes may play a protective role against dysfunctional mitochondria [49]. Another way to reinforce efficacy is to modify cargoes in MSC-EVs. It has been discovered that modifying EVs to transport certain microRNAs and drugs is a more effective means of treating liver disease [70]. However, further research needs to be done on how to make the modified EVS perform these functions.

To date, as we stated above, only a few studies have reported the application of modified MSCs to target mitochondrial dysfunction in liver disease. Improved efficacy by restoring mitochondrial function has also been found in MSC therapeutic strategies for other diseases. For example, research into cerebral injury indicated that overexpressing miR-21 in exosomes enhanced the neuroprotective effects of MSCs, which modulated mitophagy in neurocytes by directly targeting and inhibiting PTEN [71]. Therefore, the same ideas can be applied to optimize liver disease treatment by targeting dysfunctional mitochondria.

## Conclusions

In this review, we summarized the common types of mitochondrial dysfunction in liver disease and the related mechanisms. Then, we focused on the therapeutic effects of MSCs on liver diseases by modifying mitochondrial function. Interestingly, some researchers have recently turned their attention to cell-free therapies using EVs from MSCs. This method avoids some shortcomings of MSC transplantation. However, whether EVs can act as efficiently as MSCs and their role in protecting mitochondria are unclear. Finally, we noticed that the modification of MSCs is popular and has a huge advantage in treating liver disease. Most researchers tend to use genetic modifications to gain long-term, targeted, and reliable effects. However, the potential tumorigenic risks of transplanting modified MSCs have raised concerns about their safety [72]. Another concern is whether the crosstalk between cancer cells and MSCs through mitochondrial exchange is beneficial or harmful. MSCs can directly donate mitochondria to rescue injured cells. However, this altruistic behavior also benefits cancer cells because tumor growth and motility require mitochondria [53]. Therefore, further investigations into modifying MSC therapy to address these concerns are needed. In conclusion, the recent advances we reviewed in this paper are expected to provide new directions and strategies for improving the efficacy of mitochondria-targeted MSC treatment in liver disease.

## Data Availability

Not applicable.

## Abbreviations

ALD: Alcohol-associated liver disease
ATP: Adenosine triphosphate
DILD: Drug-induced liver disease
Drp1: Dynamin-related protein 1
ECT: Electron transport chain
ER: Endoplasmic reticulum
EVs: Extracellular vesicles
FFA: Free fatty acid
FIS1: Fission 1
GSH: Glutathione
HBV: Hepatitis B virus
HCV: Hepatitis C virus
HCC: Hepatocellular carcinoma
H_2_O_2_: Hydrogen peroxide
I/R: Ischemia/reperfusion
IP_3_R: Inositol 1,4,5-trisphosphate receptors
JNK: C-jun N-terminal kinase
MFN1: Mitochondrion fusion-related protein 1
MFN2: Mitochondrion fusion-related protein 2
MnSOD: Manganese superoxide dismutase
MPTP: Mitochondrial permeability transition pore
MSCs: Mesenchymal stem cells
mtDNA: mitochondrial DNA
MVs: microvesicles
NAPQI: N-acetyl-p-benzoquinone imine
O_2_• –: superoxide anion radical
OMM: outer mitochondrial membrane
OPA1: optic atrophy 1
OXPHOS: oxidative phosphorylation
PINK1: PTEN-induced putative kinase protein 1
ROS: reactive oxygen species
TCA: tricarboxylic acid
TNTs: Tunneling nanotubes
